# Modulation of Differentiation and Bone Resorbing Activity of Human (Pre-) Osteoclasts After X-Ray Exposure

**DOI:** 10.3389/fimmu.2022.817281

**Published:** 2022-05-04

**Authors:** Denise Eckert, Felicitas Rapp, Ayele Taddese Tsedeke, Daniela Kraft, Isabell Wente, Jessica Molendowska, Sidra Basheer, Markus Langhans, Tobias Meckel, Thomas Friedrich, Anna-Jasmina Donaubauer, Ina Becker, Benjamin Frey, Claudia Fournier

**Affiliations:** ^1^ Department of Biophysics, Gesellschaft für Schwerionenforschung (GSI) Helmholtzzentrum für Schwerionenforschung, Darmstadt, Germany; ^2^ Department of Macromolecular and Paper Chemistry and Membrane Dynamics, Technical University Darmstadt, Darmstadt, Germany; ^3^ Translational Radiobiology, Department of Radiation Oncology, Universitätsklinikum Erlangen, Friedrich-Alexander-Universität Erlangen-Nürnberg (FAU), Erlangen, Germany; ^4^ Department of Radiation Oncology, Universitätsklinikum Erlangen, Friedrich-Alexander-Universität Erlangen-Nürnberg (FAU), Erlangen, Germany

**Keywords:** osteoclastogenesis, inflammation, musculo− skeletal disorders, low-dose radiotherapy, x-ray, NFATc-1, chronic degenerative and inflammatory diseases

## Abstract

Low-dose radiotherapy (LD-RT) is a local treatment option for patients with chronic degenerative and inflammatory diseases, in particular musculoskeletal diseases. Despite reported analgesic and anti-inflammatory effects, cellular and molecular mechanisms related to osteoimmunological effects are still elusive. Here we test the hypothesis that X-irradiation inhibits the differentiation of precursor osteoclasts into mature osteoclasts (mOC) and their bone resorbing activity. Circulating monocytes from healthy donors were isolated and irradiated after attachment with single or fractionated X-ray doses, comparable to an LD-RT treatment scheme. Then monocytes underwent *ex vivo* differentiation into OC during cultivation up to 21 days, under conditions mimicking the physiological microenvironment of OC on bone. After irradiation, apoptotic frequencies were low, but the total number of OC precursors and mOC decreased up to the end of the cultivation period. On top, we observed an impairment of terminal differentiation, i.e. a smaller fraction of mOC, reduced resorbing activity on bone, and release of collagen fragments. We further analyzed the effect of X-irradiation on multinucleation, resulting from the fusion of precursor OC, which occurs late during OC differentiation. At 21 days after exposure, the observation of smaller cellular areas and a reduced number of nuclei per mOC suggest an impaired fusion of OC precursors to form mOC. Before, at 14 days, the nuclear translocation of Nuclear Factor Of Activated T Cells 1 (NFATc1), a master regulator of osteoclast differentiation and fusion, was decreased. In first results, obtained in the frame of a longitudinal LD-RT study, we previously reported a pain-relieving effect in patients. However, in a subgroup of patients suffering from Calcaneodynia or Achillodynia, we did not observe a consistent decrease of established blood markers for resorption and formation of bone, or modified T cell subtypes involved in regulating these processes. To assess the relevance of changes in bone metabolism for other diseases treated with LD-RT will be subject of further studies. Taken together, we observed that *in vitro* X-irradiation of monocytes results in an inhibition of the differentiation into bone-resorbing OC and a concomitant reduction of resorbing activity. The detected reduced NFATc1 signaling could be one underlying mechanism.

## Introduction

In chronic degenerative and inflammatory diseases such as Rheumatoid arthritis or Osteoarthritis, one hallmark is bone degradation and destruction of joint structures (1, 2), and in others, i.e. Calcaneodynia or Achillodynia, both degradation and neo-formation of bone can occur (3). This leads to significant pain, often to inflammation (4) and a reduction in quality of life of the patients. Standard treatments often involve non-steroidal anti-inflammatory drugs (NSAIDs), glucocorticoids, non-steroidal antirheumatics (NSARs) or biologicals like antibodies blocking inflammatory pathways. These drugs are important treatment modalities but can have severe side effects, especially when there is a need for taking them over longer periods (5, 6). As an alternative, patients with chronic degenerative and inflammatory diseases have been undergoing low-dose radiation therapy (LD-RT) for decades and report amelioration of their symptoms (7, 8). The molecular mechanisms which are responsible for this pain reduction are not yet fully unraveled, but, based on data obtained after radon exposure of patients, an influence on the immune system and bone metabolism is anticipated (9, 10).

In healthy bone tissue, a highly regulated homeostasis is maintained (11). The bone matrix is constantly remodeled by a tight interplay of bone-forming osteoblasts, sustaining osteocytes and bone resorbing osteoclasts (OC) (12). If this balance is disturbed and bone resorption is upregulated, as in chronic degenerative and inflammatory diseases, bone loss is the result, and the physiological function of joints is lost, leading to pain and movement restriction. Our hypothesis is that LD-RT has an impact on bone metabolism by restoring homeostasis transiently.

Unlike high-dose radiation therapy, as it is used in cancer therapy, for LD-RT, typically, a total dose of 3 to 5 Gy is administered locally in fractionated doses of 0.5 Gy each in 6 to 10 consecutive treatment sessions. Higher doses of X-irradiation have been reported to damage bone, such as growth disturbances in children (13, 14), radio-osteonecrosis (15) and resulting treatment-induced bone loss (CTIBL) (16) with a higher risk of fractures (17). As demonstrated for high doses in rodent models, damage to bone-forming cells, vasculature, or an impaired hormone balance can be responsible for the loss of bone mass. This is caused by inflammation, a restricted nutrient supply and a reduced bone neoformation (14, 18). For lower doses, no such harmful effects have been anticipated (19). In contrast, low-dose X-irradiation has been shown to promote osteoblast proliferation (20) and bone mineralization after fractures (21). The beneficial effect of LD-RT on inflamed joints and other parts of the musculoskeletal system is considered to be due to a modulation of anti-inflammatory signaling molecules such as iNOS (22) and a reduction of pro-inflammatory cytokines (23), which may act on a local and a systemic level of the body. To investigate the potential contribution of LD-RT to (24) modulations in bone metabolism, we aimed at investigating direct radiation induced effects on bone resorbing osteoclasts and their progenitor cells.

Mature OC (mOC) are multinucleated cells which are formed by the fusion of mononuclear progenitors of the monocyte/macrophage family and have distinct morphological features and functions (25). During this process, monocytes first differentiate under the influence of macrophage colony stimulating factor (M-CSF) into pro-osteoclasts (proOC), and then into OC precursors through receptor activator of nuclear factor ‘kappa-light-chain-enhancer’ of activated B-cells ligand (RANKL) signaling. Established markers for the differentiation stages are specific morphological features, Tartrate Resistant Acid Phosphatase (TRAP 5b) activity and fusion of three or more precursor OC, resulting in multinucleated (three or more nuclei), bone resorbing mOC. An additional marker for OC activity is the formation of a cytosolic actin ring (24, 26–28). Bone resorption as a functional marker is detectable by the formation of resorption pits on bone and the release of collagen fragments (CTX) by mOC. On a molecular level, multiple factors tightly regulate OC differentiation. Nuclear factor of activated T cells cytoplasmic-1 (NFATc1) is a master regulator of osteoclast differentiation (27, 29), regulating the expression of genes involved in cell fusion and activation of mOC (30, 31).

In most *in vitro/ex vivo* studies on OC, monocytes are isolated from blood (murine or human) and differentiated into OC by adding bone-specific growth factors, RANKL and M-CSF to the culture medium (25). Monocytic cell lines (RAW 264.7) are often used, which have some limitations because of their tumor component (32). A straightforward approach is to seed the monocytes on a conventional plastic substrate (24, 33). Then, the monocytes adhere to plastic, while other blood cells are removed by washing. The differentiated OC can then be analyzed *via* microscopy. However, for an analysis of the resorbing activity, the cultivation on plastic is not suitable. Therefore, in this study, we decided to cultivate, irradiate and differentiate OC precursors on bone slices, their physiological substrate, allowing the straightforward assessment of common markers of OC activity (e.g. CTX release and resorption pits formation). In addition, we established a method to evaluate nuclear translocation of NFATc1 in OC grown on bone by confocal microscopy. To characterize OC activity after irradiation, we quantified OC precursor and mOC according to (26), as well as the formation of resorption pits on bone and the release of collagen fragments. Furthermore, we evaluated specific cellular and molecular markers, i.e. the number of nuclei and the size of the cellular area, TRAP 5b activity and nuclear translocation of the key transcription factor NFATc1 (24, 34, 35).

We set out to verify these findings *in vivo.* Patients with musculoskeletal diseases (mostly Calcaneodynia, Achillodynia and others) underwent LD-RT at the University Hospital Erlangen, Germany and in the frame of the prospective IMMO-LDRT01 study (NCT02653079), blood was drawn and analyzed at several time points before and after therapy.

We have reported in an interim analysis of this study that after local LD-RT, pain perception of the patients, evaluated with the VAS scale (1–10), significantly decreased over time, starting from a general pain level indicating medium to severe initial stages (36) and suggesting that inflammation might be involved in the course of the diseases (3). In addition to pain reduction, some immune cell types were modulated and their activation was downregulated (36).

As in an arthritic mouse model, it has been demonstrated that local LD-RT ameliorates bone resorption in arthritic joints (37), we tested a broader spectrum of markers of bone metabolism in blood samples of a subgroup of the patients, i.e. Calcaneodynia and Achillodynia patients, enrolled in the IMMO-LDRT01 study. Although bone degradation, but also bone formation and inflammation can occur in the course of these diseases, we could not consistently confirm the involvement of changes in bone resorption and formation *in vivo* for all markers tested. We speculate that this is related to the specific diseases of the patients of this subgroup.

## Material and Methods

The supplement includes a graph to illustrate the flow of the *in vitro* experiments (**Figure S1**).

### Isolation and Cultivation of OC Precursors and Differentiation Into mOC

For the isolation of human Peripheral Blood Mononuclear Cells (PBMC), buffy coats from healthy donors was either purchased from the blood donation service (Frankfurt/Main, Germany) or blood was drawn from healthy volunteers in Vacutainer tubes (BD Vacutainer^®^ blood collection tubes, BD Biosciences, Heidelberg, Germany), both with Heparin as an anticoagulant. To isolate the PBMC from buffy coats, blood was diluted 1:1 with PBS (Sigma Aldrich Chemie GmbH, Steinheim, Germany) and carefully overlaid on Ficoll-Hypaque (Merck Millipore, Darmstadt, Germany) to avoid mixing of the phases. Vacutainer tubes with an integrated gel layer were directly used for centrifugation. The centrifugation followed at 1300 xg (buffy coat)/1500 xg (Vacutainer) for 30/20 min at room temperature (RT). Centrifugation resulted in separation into different phases, where the upper phase consisted of plasma and the second phase was the interphase containing the PBMC.

Plasma was removed for cultivation and heat-inactivated for 30 min at 56°C in a water bath (Grant SUB Aqua Pro, Fisher Scientific, Schwerte, Germany). Denatured proteins were removed by centrifugation (3360 xg, 5 min). The interphase with the PBMC was collected, washed with PBS/2 % FBS (Sigma Aldrich) and centrifuged (300 xg, 8 min). To remove the remaining erythrocytes, red blood cell lysis buffer (RBC, 8.29 g NH_4_Cl, 1 g KHCO_3_, 37.2 mg Na_2_EDTA, 800 ml H_2_O, pH 7.2-7.4 in 1 L H_2_0) was added (buffy coat: 5 ml, Vacutainer: 2 ml) and incubated at RT for 5 min. Then, PBS/2 % FBS (Sigma Aldrich) was added to stop the reaction (buffy coat: filled to 50 ml, vacutainer: filled to 15 ml), cells were collected (300 xg, 8 min) and suspended in X-Vivo-15 medium (Lonza/Biozym Scientific GmbH, Oldendorf, Germany) containing 3 % autologous plasma and 1 % Penicillin/ Streptomycin (Pen/ Strep) (PAN-BIOTECH, Aidenbach, Germany) (buffy coat: 50 ml, vacutainer: 5 ml).

Afterwards, PBMC were seeded on bone slices (ids immunodiagnostics, Boldon, UK) in X-Vivo-15 medium and incubated at 37°C/5 % CO_2_ for 1-2 hours, allowing monocytes to adhere [modified after (24)]. For successful seeding on bone slices (4x10^5^ cells in at least 100 µl), the slices were first placed in 96-well plates during the adhesion process, and at the same time, non-adherent lymphocytes were removed, and the remaining attached cells were transferred to 24-well plates, containing 500 µl of differentiation medium (Alpha-medium, Merck Millipore). The medium was supplemented with 1 % Pen/Strep, 10 % autologous plasma and differentiation factors, i.e. RANK-L (40 ng/ml, EMD Millipore Corp., Billerica, MA, USA) and M-CSF (25 ng/ml, Miltenyi Biotec, Bergisch Gladbach, Germany), marking the onset of the differentiation process. Almost all attached cells displayed typical morphological features of the first OC differentiation stage (26) and were therefore classified as precursor OC from this time point on. For the following differentiation process, OC precursors were cultured for 21 days. Medium was changed twice a week, and supernatants were collected and stored at -80°C.

### Irradiation of OC Precursors

After the adhesion step and in the presence of growth factors, attached cells were exposed to X-rays at single doses of 0.5 to 10 Gy with a dose rate of 1-2 Gy/min or at fractionated doses in 4 or 10 fractions (0.5 or 1 Gy each) in 24 h intervals to simulate a therapeutic setting. Irradiation was performed at RT with an X-ray tube with 250 kV and 16 mA (Seifert Isovolt DS1, Ahrensberg, Germany). Sham-irradiated cells were also transported to the X-ray tube and used as controls.

### Analysis of Apoptotic Cells

Apoptotic cell death was evaluated *via* morphologic features of apoptosis (38–40) in osteoclast cells, such as nuclear condensation, nuclear fragmentation and membrane blebbing in the same microscopic pictures used for osteoclast characterization. The fraction of apoptotic cells was calculated in relation to the total cell number ranging from 150 to 250 cells per field of view at 6 and 12 hours and 3, 5, 7, 14 and 21 days after X-irradiation.

### Identification of Precursor and mOC by Fluorescent Triple Staining

Morphological (cell size, number of nuclei) and functionality parameters of OC (TRAP, actin ring) were investigated according to standard criteria (24, 26, 28). However, additional molecular markers of OC differentiation have not been investigated because the choice of a bone matrix for cultivation brings along lower cell numbers than plastic, limiting the possibility of performing PCR analysis.

After fixation with 3.7 % PFA (Merck KGaA, Darmstadt, Germany) for 15 min at RT cells were incubated with TRAP staining solution for 10 min at 37°C. The staining solution consists of TRAP buffer (0.1 M acetate buffer: 35.2 ml 0.2 M Sodium Acetate solution, 14.8 ml 0.2 M acetic acid, 50 ml; Millipore, Burlington, USA) mixed with 0.3 M Sodium Tartrate (pH 5.5), 10 mg/ml Naphthol AS-MX phosphate, Triton X-100, 0.3 mg/ml Fast Red Violet LB Salt (all from Sigma Aldrich Chemie GmbH). After two washes with PBS, FITC-Phalloidin (0.66 µg/ml in PBS) (Sigma Aldrich Chemie GmbH) was added for 25 min in the dark at RT. Next, DAPI (1 μg/ml in PBS) (BD Biosciences) was added and incubated for another 7 minutes. After the last washing step, the cells were stored at 4°C in PBS in the dark.

**Figure 1:**
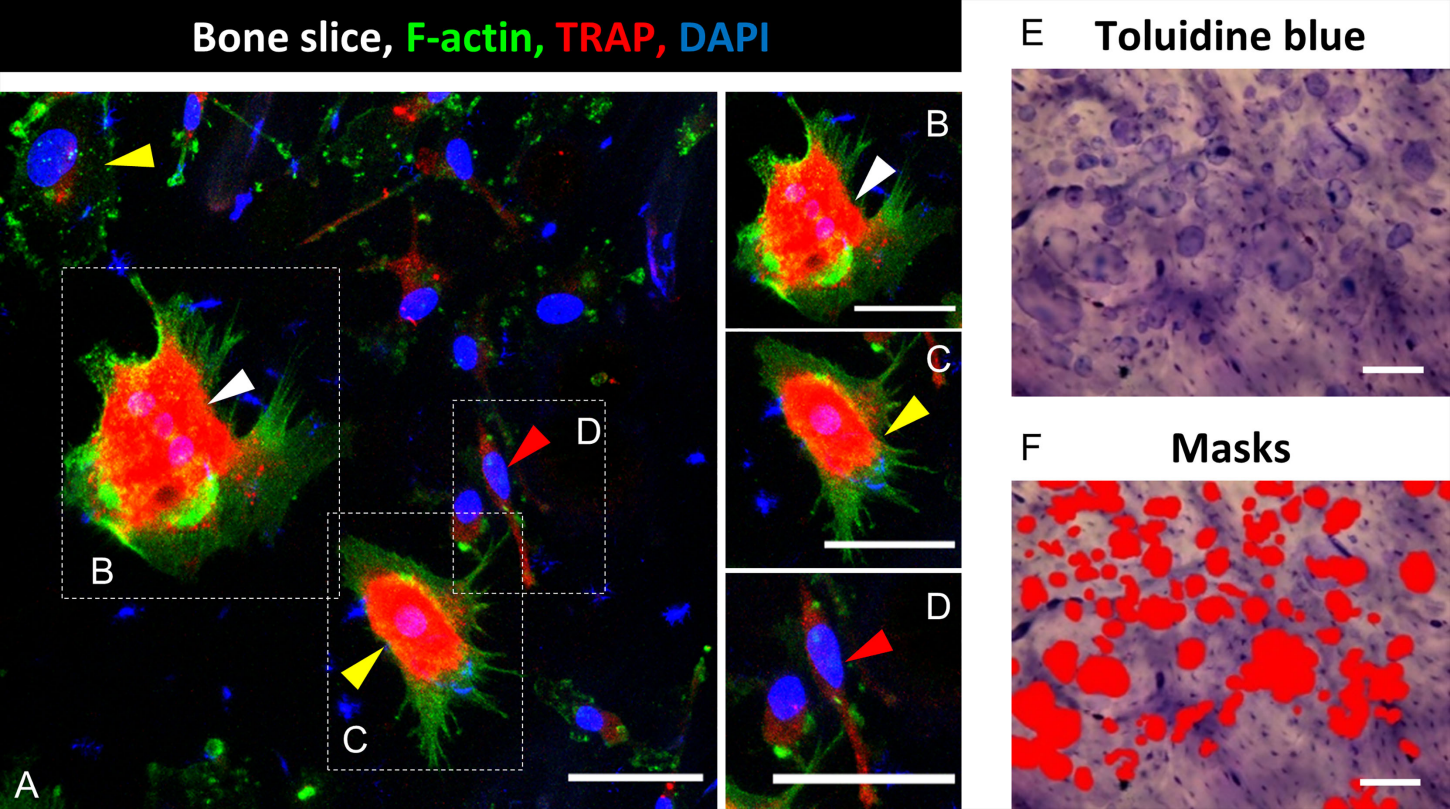
Identification of mature osteoclasts (mOC) on bone slices and quantification of resorbing activity 21 days after onset of the differentiation process. A triple staining for OC markers was applied (TRAP, red; F-actin, green; nuclei: blue; **(A)**. A cell was classified as mOC when it TRAP-positive and had ≥ 3 nuclei (white arrowheads; all mOC showed an actin ring, **(B)**, precursor (preOC, round with one or two nuclei, yellow arrowheads, **(C)**, proOC (spindle-shaped with a single nucleus, red arrowhead, **(D)**. After microscopy, cells were removed, and resorption pits were visualized by Toluidine blue staining **(E)**. Semi-quantitative image analysis was performed using ImageJ and masks to measure the resorbed areas per field of view **(F)**. Scale bar: 50 µm **(A-D)**, 100 µm **(E, F)**.

Microscopic pictures were either taken on a confocal microscope (DMI4000B, Leica, Wetzlar, Germany; 5-10 images per bone slice) or with an epifluorescence microscope (DMIRE2, Leica, 20x) (**Figure 1A**). For evaluation, ImageJ (Wayne Rasband, NIH, Bethesda, USA) was used. Cells were classified into three subgroups according to established criteria (24, 26, 28): pro- (spindle-shaped with a single nucleus, **Figure 1D**), pre- (round with one or two nuclei, **Figure 1C**) and mOC (TRAP-positive and with three or more nuclei, **Figure 1B**). Throughout the text, pro-and pre-OC are not distinguished and termed OC precursors, unless stated otherwise. We show the total number of OC (OC precursor and mOC) and the number of mOC (reported per total number of OC and as fold change compared to controls). Furthermore, mOC with at least three nuclei were morphologically evaluated with regard to the number of nuclei and the cell surface area. The mean value for the total number of OC (OC precursor plus mOC) and mOC per total number of OC, was evaluated from 5-10 images per bone slice and afterwards from the technical replicates (n=2-3) for each donor. Finally, the mean value was calculated from all donor samples (N=4-8). No monocytes or dead cells were identified on the bone slices after 21 days of cultivation.

### Measurement of Cellular Area and Counting of Nuclei per mOC

For the measurement of the cellular area of mOC, ImageJ was used. Outlines of cells with respect to actin staining (**Figures 1A–D**) were traced with the freehand tool, and the enclosed area was measured. The number of nuclei were also determined using ImageJ. An enhanced number of nuclei (three or more nuclei) is an established differentiation marker of mOC (25).

### Analysis of Bone Resorbing Activity

A functional analysis of bone resorbing activity was performed by staining with toluidine blue. Cells were first removed with ammonium hydroxide solution (0.25 M Ammonium hydroxide solution, Sigma Aldrich Chemie GmbH, diluted with dist. water) for 5 min at RT and then wiped from the bone slice with a paper tissue. Toluidine blue staining solution (Toluidine blue staining solution in citric acid: 1 % Toluidine blue (Fluka/Sigma Aldrich Chemie GmbH) solved in 1 % sodium tetraborate (40°C, soluble in H_2_O; Sigma Aldrich Chemie GmbH) was added to the bone slices for 10 min, and residues were washed away thoroughly with PBS. Images were taken with a light microscope (BX61, Olympus, Hamburg, Germany, 5-7 images per slice, 10x, **Figure 1E**) or a fluorescence microscope (Revolve 4M, Echo, San Diego, CA, USA). For analysis of the resorbed area, the color threshold function of ImageJ was used in order to mark the stained resorption pits (**Figure 1F**). Marked areas were measured and related to the total area per picture. In cases of weak staining or low contrast to the background, the ROI (Region of Interest) function was used to determine the resorbed area precisely. Per bone slice, six images were evaluated, and from this, the mean value was calculated. Subsequently, the mean value was calculated from the technical replicates of the respective healthy donors, and finally, the mean value was calculated from the respective healthy donors.

### NFATc1 Immunofluorescent Staining

For immunofluorescent staining, 8 x 10^5^ OC precursor cells were seeded on bone slices, fixed after 3, 5, 7 and 14 days of cultivation with 3.7 % PFA for 30 min at RT and washed with PBS. Then bone slices with cells were incubated with permeabilization solution (0.2 % Triton X-100, Sigma Aldrich Chemie GmbH) for 10 min at RT, followed by incubation in blocking buffer (5 % BSA, Sigma Aldrich and 5% Goat serum, Invitrogen) for 1 h. Afterwards, bone slices were incubated with anti-NFATc1 (Santa Cruz, Dallas, TX, USA, 1:200) overnight at 4°C. The next day, after washing three times with PBST (0.1 % Triton X-100, Sigma Aldrich Chemie GmbH), bone slices were incubated for 2 h with secondary antibody goat anti-mouse Alexa Fluor 488 or goat anti-mouse Alexa Fluor 647 (Thermo Fisher, Waltham, MA, USA, 1:500 in PBST) at RT. Subsequently, the bone slices were washed three times with PBS-T, TRAP staining buffer was added for 10 min at 37°C, and nuclei were counterstained with DAPI (Sigma Aldrich Chemie GmbH). Images were captured using a confocal microscope (DMIRE 4000B or TCS SP8, Leica) with a 20x or 40x oil-immersion objective. Fluorescent intensity of NFATc1was calculated using ImageJ from the integrated density of the entire cell, minus the integrated density of the nucleus. Subsequently, the values for nucleus or cytoplasm were divided by the total intensity of the cell.

### Study Design of the IMMO-LDRT01 Trial

For details on the IMMO-LDRT01 trial (NCT02653079), see (36). Briefly, all patients enrolled were affected by chronic degenerative and inflammatory diseases. They have signed an informed consent, the trial was ethically approved by the ‘Ethik Kommission der Friedrich-Alexander-Universität Erlangen-Nürnberg’ (#289_15 B) and followed the ‘Declaration of Helsinki’ in its current form. Patients underwent a 1^st^ series of irradiation with X-rays over 3 weeks, i.e. 6 fractions with a single dose of 0.5 Gy each. After 3 months, a 2^nd^ series with the same scheme was administered if the patient stated that the therapeutic outcome was not satisfying. For the study presented here, blood samples have been drawn from a subgroup of 22 patients before (tp1) and after (tp2) the 1^st^ series, before (tp3) and after (tp4) the 2^nd^ series and 3 months later at the final follow-up appointment (tp5). The patients of this subgroup suffered mostly from Calcaneodynia and Achillodynia (see **Table 1**). According to their personal decision, all patients underwent in this study the 1^st^ and the 2^nd^ series. The pain perception of the patients, evaluated with the VAS scale (1–10), showed a significant decrease over time, starting from a general pain level of 6.5 and dropping to 3.8 (36). This mean pain level indicates advanced stages, suggesting that inflammation might be involved in the course of the treated diseases.

**Table 1:** Characteristics of patients (IMMO-LDRT01 trial).

Total number		22	patients
Age at start	Mean	60	Years
	Range	38-77	Years
Gender	Male	4	patients
	Female	18	patients
Indications	Calcaneodynia	14 (64 %)	patients
	Achillodynia	4 (18 %)	patients
	Others	4 (18 %)	patients

In the leukocyte fraction of the blood samples, the ratio of Th17/Treg was determined. In addition, in the plasma or serum fraction, resorption markers (CTX, TRAP 5b) and formation (OCN) of bone were quantified.

### Analysis of Th17/Treg by Flow Cytometry

For the analysis of Th17/Treg ratio, isolated PBMC from LD-RT patients (see *Isolation and Cultivation of OC Precursors and Differentiation Into mOC*) were used. After isolation, 1x10^6^ cells were fixed with 1x Human FoxP3 Buffer A (BD Pharmingen™, BD Pharmingen, Heidelberg, Germany) for 20 min at RT in the dark. Afterwards, they were centrifuged at 500 xg for 5 minutes and washed with PBS + 2 % FCS (500 xg, 5 min). Subsequently, cells were permeabilized by adding 0.5 ml of 1x working Human FoxP3 Buffer C (BD Pharmingen™) for 30 min at RT in the dark. Following, the cells were washed twice with PBS + 2 % FCS (500 xg, 5 min). Finally, fixed and permeabilized cells were stained with CD4 (PerCP-Cy5.5, clone SK3, #566923, BD), FoxP3 (Alexa Fluor 647, clone 259D/C7, #560045, BD), ROR-α (PE, clone #784652, #IC8924P-100, R&D Systems, Minneapolis, USA) for 40 min at RT in the dark. After two further washing steps, the expression of cell surface or intracellular markers was measured using a flow cytometer (CytoFLEX S, Beckman Coulter, Krefeld, Germany). The gating strategy is shown in **Figure 6A**. Using CytExpert software (Beckman Coulter), the frequency of cells related to the total number of CD4^+^ cells was analyzed: CD4^+^FoxP3^+^ cells were classified as Treg cells and CD4^+^ ROR-α^+^ as Th17 cells.

### Detection of CTX Fragments in Cell Culture Supernatants and Patient Samples

For the analysis of released collagen fragments, CTX ELISA [Serum CrossLaps^®^ (CTX-I) ELISAs, ids immunodiagnostics, Boldon, UK] was used for cell culture supernatants and plasma from patients according to the manufacturer’s protocol.

In brief, 50 µl of each standard, control and undiluted sample were pipetted on the Streptavidin-coated plate. Afterwards, 150 µl of the antibody solution AB SOLN were added and incubated for 120 min at RT on a microtiter plate mixer (300 rpm, Heidolph UNIMAX 1010, Schwabach, Germany). Subsequently, wells were washed 5 times with diluted wash solution. Then, 100 µl of substrate solution were added to each well and incubated for a further 15 min in the dark on a microtiter plate mixer (300 rpm). Subsequently, 100 µl of stopping solution (H_2_SO_4_) were added, and finally, the absorbance was measured with SpectraMax i3x (Molecular Devices, California, USA) at 450 nm with reference at 650 nm.

### Detection of TRAP 5b Activity in Serum of Patients

For analysis of TRAP 5b activity in serum of patients, BoneTRAP^®^ ELISA (ids immunodiagnostics, Boldon, UK) was used as described in the manufacturer’s protocol. In brief, 100 µl of standard, control and patient serum were pipetted in duplicates. Afterwards, 50 µl of Releasing Reagent were added to each well and were incubated with constant shaking for 1 h at RT (with foil). Then, wells were washed four times with 300 µl Wash Buffer. Subsequently, 100 µl of Substrate were added and incubated for 1 h at 37°C (with foil). After the incubation step, 25 µl of Stop Solution were added. Finally, the absorbance was measured with SpectraMax i3x (Molecular Devices, California, USA) at 450 nm with reference at 650 nm.

### Quantification of OCN in Plasma of Patients

For the analysis of Osteocalcin (OCN), Osteocalcin Human ELISA Kit (Thermo Fisher, Waltham, MA, USA) was used for plasma from patients according to the manufacturer’s protocol.

In brief, 25 µl of each standard, control and undiluted sample were pipetted on the Streptavidin-coated plate. Afterwards, 100 µl of the Anti-OST-HRP conjugated Solution were added and incubated for 120 min at RT. Subsequently, wells were washed 3 times. Then, 100 µl of Stabilized Chromogen Solution were added to each well and incubated for a further 30 min at RT. Subsequently, 100 µl of Stop Solution were added, and finally, the absorbance was measured with SpectraMax i3x (Molecular Devices, California, USA) at 450 nm with reference at 650 nm.

### Statistical Analysis and Graph Settings

**Figure 2:**
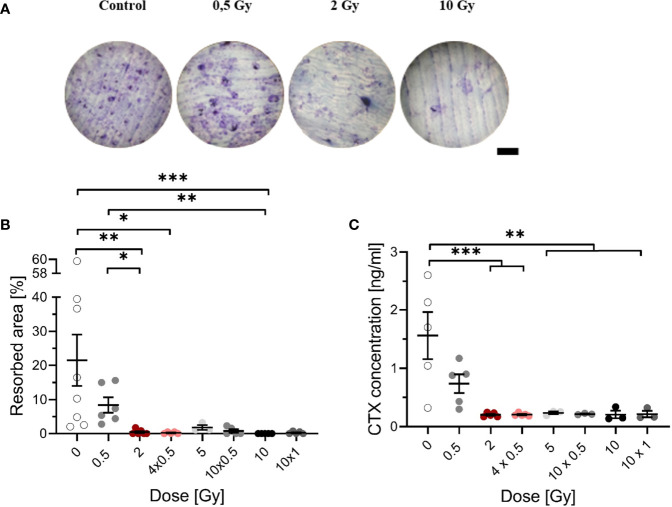
Decreased resorbing activity of mOC on bone slices at 21 days after exposure to ionizing radiation (X-rays). Monocytes were attached on bone slices, then irradiated in the presence of growth and differentiation factors (M-CSF, RANKL) with single (0.5, 2 and 10 Gy) and fractionated doses (4 x 0.5, 10 x 0.5 and 10 x 1 Gy). Resorbing activity and release of collagen fragments (CTX) were investigated at 21 days. Resorbing activity of mOC was visualized by Toluidine blue, and the area of resorbed bone was measured by ImageJ [**(A)**, scale bar 100 µm]. Bone resorption was expressed as resorbed bone area in percent of total bone per field of view **(B)**. Soluble collagen fragments (CTX) were measured in culture supernatant as markers for OC resorbing activity **(C)**. Significance was tested with Kruskal-Wallis **(B)** for non-normal distributed data and with one-way ANOVA **(C)** for normal distributed data. P-values are indicated by asterisks with * for p ≤ 0.05, ** for p ≤ 0.01, and *** for p ≤ 0.001. Error bars are reported as the mean ± SEM; N = 3-8.

**Figure 3:**
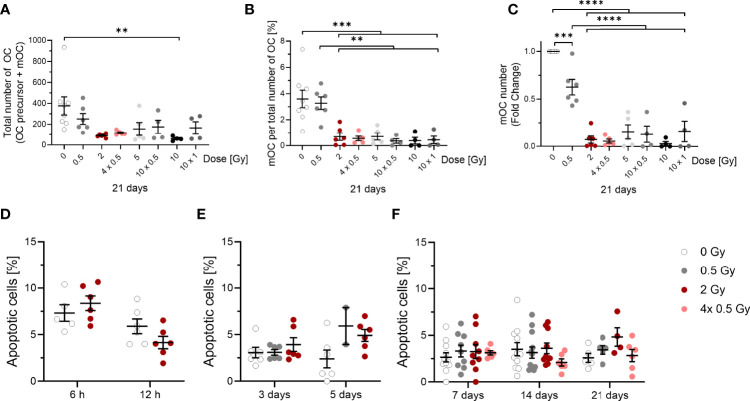
Influence of irradiation (X-rays) on the survival of OC (OC precursors and mOC) and their differentiation into mOC (in relation to the respective OC number in each sample), following cultivation on bone slices for 21 days and apoptotic frequencies between 6h and 21 days after exposure. Monocytes were attached on bone slices, then irradiated in the presence of growth and differentiation factors (M-CSF, RANKL) with single doses (0. 0.5 and 2 Gy) and fractionated doses of X-rays (4× 0.5, 10x0.5 and 10x1 Gy). Differentiation into OC precursor and mOC was assessed at 21 days of cultivation. The total number of OC per field of view (OC precursor + mOC) is decreased in a dose-dependent manner **(A)**. On top of this reduction, mOC per total number of OC in each sample decreased after irradiation **(B)**. The fold change of mOC number compared to controls also shows a decrease **(C)**. mOC were identified based on morphological patterns (TRAP+, F-actin+ with ≥ three nuclei). Apoptotic frequencies were evaluated in cells 6 and 12 hours and after 3 to 21 days after exposure to the same doses as in **(A-C)** by morphological features **(D-F)**, indicating very low apoptotic frequencies at all time points investigated. Significance was tested with Kruskal-Wallis **(A, E, F)** for non-normal distributed data and with one-way ANOVA **(B, C)** for normal distributed data for more than two groups and with t-test **(D)** for normal distributed data for two groups. P-values are indicated by asterisks with *** for p ≤ 0.001 and **** for p ≤ 0.0001. Error bars are reported as mean ± SEM; N = 3-8.

**Figure 4:**
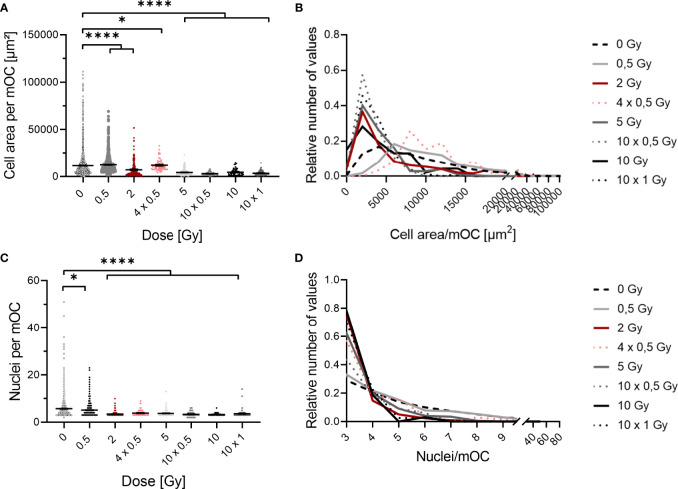
Fusion of OC precursors is inhibited by irradiation with X-rays. At 21 days after exposure, mOC (21d in culture) exhibit a smaller area on bone slices after irradiation **(A)**. Depiction of the distribution of values demonstrates a shift from unirradiated cells and cells irradiated with 0.5 Gy with a higher area to cells with a smaller area irradiated with doses of 2 Gy or more **(B)**, bin size = 2000. mOC possess a smaller mean number of nuclei per cell after irradiation **(C)**, while the distribution of values shows the shift to the minimum number of three nuclei per mOC at doses higher than 2 or more Gy **(D)**, bin size = 1. Area measurements were performed by F-actin staining, nuclei were counterstained with DAPI (≥ three nuclei), and confocal microscopy pictures were evaluated using ImageJ. Significance was tested with Kruskal-Wallis for non-normal distributed data **(A, C)**. P-values are indicated by asterisks with * for p ≤ 0.05 and **** for p ≤ 0.0001. Error bars are reported as the mean ± SEM; N = 4-8.

**Figure 5:**
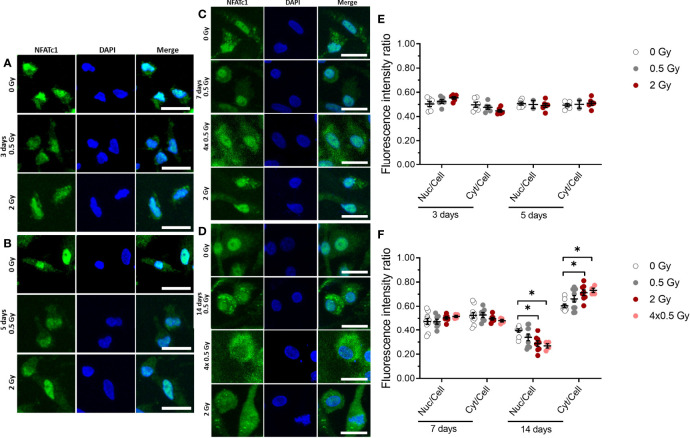
NFATc1 translocation into the nucleus is partially inhibited by ionizing radiation (X-rays) at a later stage (14 days) of OC differentiation. Monocytes were attached on bone slices, then irradiated in the presence of growth and differentiation factors (M-CSF, RANKL) with single doses (0, 0.5 and 2 Gy) and fractionated doses of X-rays (4× 0.5 Gy). Immunostaining of NFATc1 protein (green) showing cytoplasmic and nuclear localization in osteoclast and counterstain with DAPI (blue) **(A-D)**. NFATc1 translocation into the nucleus was quantified using ImageJ software. The graph represents the fluorescent intensity ratio of the nucleus or cytoplasm to the cell at 3 and 5 days of cultivation **(E)** and 7 and 14 days of cultivation **(F)**. Activated NFATc1 is translocated from the cytoplasm into the nucleus **(E)**. Nuclear translocation is inhibited in the 4× 0.5 and 2 Gy irradiated samples at 14 days after exposure **(F)**. Significance was tested with Kruskal-Wallis for non-normal distributed data. P-values are indicated by asterisks with * for p ≤ 0.05. N = 2-5. The values are given as mean ± SEM from at least 100-200 cells per donor. Scale bar: 20 µm **(A-D)**.

**Figure 6:**
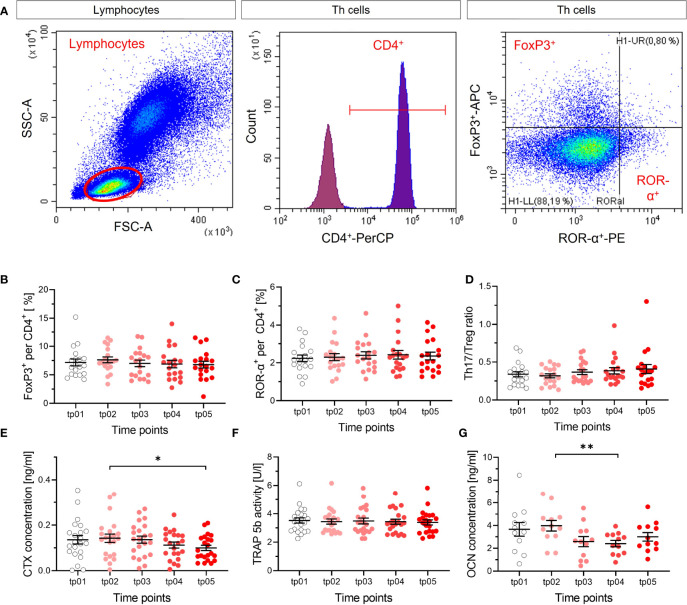
No apparent modification of T cell subtypes and markers of resorption and formation of bone. The fractions of Treg (FoxP3^+^) **(B)** and Th17 (ROR-α^+^) **(C)** per and CD4^+^ cells were measured in blood by flow cytometry before the start of LD-RT (tp01) and at several time points during and after therapy (tp02-tp05; N = 22). The ratio of both cell types **(D)** was calculated. Representative dot plots and gating strategy are shown in **(A)**. Lymphocytes were determined in the SSC/FSC dot plot. After exclusion of doublet’s, CD4^+^ (Per-CP) T helper cells were identified in the histogram. Subsequently, Treg cells were classified as FoxP3^+^ (APC) and Th17 cells as ROR-α^+^(PE) from CD4^+^ cells. No changes in Treg cells, Th17 cells, nor Th17/Treg ratio are detectable after LD-RT, as for the markers of resorption and formation of bone, indicating no impact on T cell subtypes that are involved in the regulation of bone metabolism. CTX **(E)**, TRAP 5b **(F)** and OCN **(G)** were measured (tp01-tp05; N = 22). CTX concentration decreases slightly over time, which is not reflected by the activity of TRAP 5b, and OCN concentration decreases. Significance was tested with RM one-way ANOVA **(E, G)** for normal distributed data and Friedman Test **(B-D, F)** for not-normal distributed data. P-values are indicated by asterisks with * for p ≤ 0.05 and ** for p ≤ 0.01.

Data and statistical analyses were calculated with GraphPad Prism 8 (GraphPad Software, Inc., La Jolla, CA, USA) and are displayed with mean ± SEM. Testing for normal distribution was done by performing the Shapiro-Wilk test. For unpaired and normal distributed data with more than two groups, one-way-ANOVA followed by a posthoc Tukey’s multiple comparison test (**Figures 2C**, **3B, C**) was applied. For unpaired and non-normal distributed data with more than two groups, the Kruskal-Wallis test followed by Dunn’s test (**Figures 2B**, **3A, E, F**, **4A, C**, **5E, F**) was applied. For unpaired and normal distributed data with two groups t-test was applied (**Figure 3D**). For analysis of patient samples (paired with more than two groups), repeated measures one-way ANOVA was used, followed by posthoc Tukey’s multiple comparison tests (**Figures 6E, G**) for normal distributed data, or Friedman Test (**Figures 6B–D, F**) for non-normal distributed data. P-value ≤ 0.05 was considered as significant.

Histograms of data distribution are depicted for cell area and number of nuclei in **Figure 4**. Data were normalized to values of unirradiated cells, and a proper bin size was applied (for area bin = 2000 µm and nuclei bin = 1).

## Results

### X-Ray Irradiation of Osteoclast Precursors Cultivated on Bone Slices Decreases Resorbed Area and CTX Fragment Release in a Dose-Dependent Manner

As shown earlier, LD-RT decreases pain in patients with chronic degenerative and inflammatory diseases, which is, at least for Rheumatoid arthritis (RA), thought to be related to reduced activation of OC (41). To investigate this, we examined the impact of single and therapy-relevant fractionated X-ray doses on the formation of resorption pits by OC, that underwent culture on bone slices during 21 days after exposure (exemplary images are shown in **Figure 2A**). Irradiation induced a decrease of resorbed area, dropping sharply with increasing dose (**Figure 2B**). The resorbed area decreased more than 3-fold after irradiation with 0.5 Gy compared to unirradiated samples. After irradiation with 2 Gy or more, resorbed area was clearly reduced to a very low level, for single as well as for fractionated doses.

To confirm the obtained results, an additional marker of bone resorption, the release of CTX into the supernatant was measured. A dose-dependent decrease in the CTX concentration was found (**Figure 2C**). After irradiation with 0.5 Gy, the CTX concentration was reduced by a factor of 2 compared to unirradiated samples; after exposure to single doses of 2 - 10 Gy and fractionated doses of 4 x 0.5 Gy, 10 x 0.5 Gy and 10 x 1 Gy, the concentration of CTX was significantly decreased, at least by ~7-fold. The results indicate that the bone-resorbing activity is already reduced after exposure to a single dose of 0.5 Gy.

### The Fraction of mOC Is Decreased After X-Ray Irradiation on Bone Slices

To address whether a decreased cell number is responsible for the reduced bone resorbing activity of OC after irradiation, we next investigated 21 days after exposure the total OC number (OC precursor and mOC). In order to discriminate between cell loss over all differentiation stages and an inhibition of terminal differentiation, we report the respective fraction of mOC relative to the total number of OC and, in addition, the fold change of the number of mOC compared to controls. Monocytes were only sporadically observed and exclusively until 3 days after exposure.

The total number of OC, including proOC, preOC and mOC, was not significantly decreased at a single X-ray dose of 0.5 Gy, but decreased at single or fractionated doses of 2 Gy or higher (around 2.5-fold for 2, 4x 0.5, 5 and 10x 0.5 Gy, **Figure 3A**). A single dose of 10 Gy even lowered the cell number around 4-fold, whereas the fractionated dose of 10x 1 Gy resulted in a level comparable to the other doses.

Notably, on top of cell loss, the fraction of mOC, normalized on the respective total number of OC that was detectable for each sample, was strongly reduced after irradiating with X-ray in a dose-dependent manner. After 0.5 Gy, almost no change was detected, but after exposure to single or fractionated doses equal to or exceeding 2 Gy, a strong and significant reduction of mOC was visible (~5-fold for 2 and 4x 0.5 Gy, ~3-fold for 5 Gy, ~5-fold for 10 x 0.5 Gy, ~10-fold for 10 Gy and ~4-fold for 10x 1 Gy; **Figure 3B**). This indicates an impact of irradiation with single or fractionated doses on OC differentiation that is not detectable for a single dose of 0.5 Gy, but for all doses exceeding 0.5 Gy. The fold change of the number of mOC shows a clear decrease, significant for doses equal to or exceeding 0.5 Gy (**Figure 3C**).

To analyze the observed cell loss in more detail, we addressed apoptosis of OC precursor as a potential reason for our findings. For this, we analyzed microscopic pictures for morphological markers of apoptosis, like condensed or fragmented nuclei. At these earlier stages of differentiation (6 and 12 hours), there was a background level of cell death of around 4-8 % (**Figure 3D**). After 3 to 21 days, the level of apoptotic cells in irradiated samples did not exceed 5 %, while the level in unirradiated samples stayed at around 3 % (**Figures 3E, F**). Apoptosis did not increase after irradiation, most likely due to the protective effect of the differentiation factors (42). Therefore, apoptosis is unlikely to account for the drastic changes in mOC fractions reported above.

Directly after exposure, the induction of apoptosis in monocytes was assessed separately, without adding differentiation factors. The apoptotic frequency turned out to be low (see **Figure S2**) and was not further investigated in our study.

### Cell Fusion Is Inhibited After X-Ray Irradiation

Based on the finding that the fraction of mOC is strongly reduced after irradiation, we set out to identify the underlying mechanism. During differentiation, OC precursors fuse to form mOC, characterized by possessing three or more nuclei (43). As this was observed 21 days after irradiation and loss of OC cell explains only in part the massive reduction of resorbing activity, we analyzed next if the fusion of precursors is altered by X-ray irradiation. To this end, we analyzed the area covered by a single mOC and the average number of nuclei per mOC by fluorescent staining for OC markers TRAP, F-actin and nuclei (**Figure 4**).

X-ray irradiation reduced the area covered by mOC in a dose-dependent manner (**Figure 4A**). After irradiation with a single dose of 0.5 Gy, the mean cell area was only marginally changed, while it was decreased by a fold-change of ~1.5 after a single dose of 2 Gy. When this dose was delivered in fractions (4x 0.5 Gy), the mean cell area was only slightly changed compared to the single dose of 2 Gy. After single doses of 5 or 10 Gy, or fractionated doses of 10x 0.5 Gy or 10x 1 Gy, the area was strongly reduced by around 2.5- to 3-fold. This effect is even clearer when looking at the distribution of values in the histogram (**Figure 4B**). The distribution of the values for the area of unirradiated or 0.5 Gy-irradiated cells is wide and unimodal, whereas for higher doses, the distribution is more right-skewed, peaking at lower areas. Notably, the curve for the single dose of 2 Gy resembles those of higher doses, whereas the distribution of 4x 0.5 Gy is wider, bimodal and more comparable to the single dose of 0.5 Gy.

The mean number of nuclei per mOC did not change after a single dose of 0.5 Gy (**Figure 4C**), but was clearly reduced for single and fractionated delivery of doses exceeding 0.5 Gy. A strong reduction was measured after a single dose of 2, 5 and 10 Gy (~2-fold) and also after fractionated doses of 4x 0.5, 10x 0.5 and 10x 1 Gy (~2-fold). In the depiction of distributions, it becomes evident that after doses of 2 Gy or higher, a shift towards the minimum number of nuclei identifying an mOC occurs (**Figure 4D**). While histograms for all doses are right-skewed, the distribution is flatter for unirradiated or 0.5 Gy-irradiated cells, since a larger fraction of these cells has more than three nuclei, reaching up to 20 (after 0.5 Gy) or even 50 nuclei (unirradiated). These findings point to radiation induced alterations occurring during fusion in the differentiation process.

### X-Ray Irradiation Partially Inhibits Nuclear Translocation of NFATc1 Protein

Our data suggest that the fusion of OC precursor is affected by ionizing irradiation. So far, it is known that NFATc1 is a master regulator of osteoclast differentiation and fusion (27, 44), and dephosphorylation of cytosolic NFATc1 by the phosphatase calcineurin induces translocation of the protein into the nucleus. Therefore, we set out to investigate the nuclear translocation of NFATc1 protein in M-CSF and RANKL-stimulated OC populations, which were irradiated exemplarily for single doses of 0.5 and 2 Gy and a fractionated dose of 4×0.5 Gy and assessed after 3, 5, 7 and 14 days of cultivation on bone slices. As shown in **Figure 5**, at early stages (up to 7 days), immunofluorescence staining demonstrated that NFATc1 protein levels in the nucleus are not affected significantly upon ionizing irradiation (**Figures 5A, B, E**). However, at a later stage (14 days) of osteoclastogenesis, nuclear translocation of the NFATc1 protein still remains unchanged for 0.5 Gy, but is significantly reduced after a single dose of 2 Gy or fractionated delivery of the same dose (4×0.5 Gy) of X-ray irradiation (**Figures 5C, D, F**).

### Markers for Changes in Bone Metabolism Are Not Modified *In Vivo* by LD-RT treat Calcaneodynia and Achillodynia Patients

In the frame of the IMMO-LDRT01 trial [details in (36)], blood samples were taken at several time points before and after therapy from a subgroup of patients, mostly suffering from Calcaneodynia and Achillodynia and further analyzed. In our analysis, we focused on the ratio of subtypes of T cells that impact bone metabolism, i.e. Th17 and Treg cells, the release of CTX in plasma samples, and the activity of the bone resorbing enzyme TRAP 5b, as well as a marker for bone formation OCN (**Figure 6**). As can be inferred from **Figures 6A–D**, the ratio of Th17 and Treg cells is not significantly changed after LD-RT, indicating no impact of these T cell subtypes on inflammation and/or bone metabolism after treatment. In **Figures 6E, F**, the results for bone resorption markers are shown, revealing a weak statistical significance for a decrease in CTX, but no detectable impact of LD-RT on TRAP 5b. The results for the marker of bone formation, **Figure 6G**, show a small but significant decrease between two time points. Taken together, at least for Calcaneodynia and Achillodynia and based on the markers tested after LD-RT, no clear and consistent impact can be inferred from our *in vivo* data.

## Discussion

The goal of the study presented here was to test the hypothesis, that one important mechanistic basis of pain relief and anti-inflammatory effects of local exposure to low X-ray doses in LD-RT is a transient inhibition of the terminal differentiation and activity of bone resorbing human OC. To investigate this, the experiment was designed to be as close as possible to the treatment situation in LD-RT by cultivating OC precursor on bone slices, which descended from monocytes that were isolated from peripheral blood from healthy donors and then cultivated with OC differentiation factors. In addition, X-ray irradiation schemes resembling those used in LD-RT for patients were applied. While in other studies the administration of low X-ray doses in LD-RT is not reflected, i.e. rodent cells or cell lines are used (33, 45), or mice were subjected to whole-body exposure (46, 47); our study is a new approach. After all, beside differences in the experimental design, the conflicting results may be reconciled by assuming a time effect, i.e. an enhanced osteoclastogenesis early after irradiation and reduced osteoclast activity at later times after exposure (37, 46).

Using established markers for bone resorption, such as the release of CTX or resorbed area (48, 49), we detected significant reductions for both markers after exposure (**Figure 2**). This is in line with the significant reduction of fractions of mOC as well as the fold change of number of OC related to the controls, which is not caused by apoptosis (**Figures 3** and **S2**).

Thus, we anticipate that X-irradiation inhibits the differentiation of OC precursors into mOC. This holds true also for repeated irradiations with 0.5 Gy fractions, which is similar to the LD-RT regimen. Hints for an inhibition of bone resorption by osteoclasts despite increasing cell numbers were obtained in murine mOC of healthy but not arthritic joints after X-irradiation in the dose range of 0.1-0.5 Gy (19). This interesting observation we cannot confirm, but our findings are in line with the observed decrease in the number of mOC from mouse arthritic joints, in spite of differences in the experimental approach, i.e. species, time points of analysis, and cultivation on bone versus plastic.

Interestingly, pharmacological downregulation of ROS leads to reduced numbers of mature osteoclasts as well as decreased resorbing activity in the remaining OC *via* RANKL/NFATc1 signaling without cytotoxicity (29, 50, 51). As the intracellular ROS level is reduced by exposure to low doses of X-rays (52), this reduced level of oxidative stress could be one cause for the reduced resorbing activity of irradiated OC precursors in our study. In line with this, results are reported in several preclinical rodent studies [(37, 53, 54), reviewed in (7)], which support radiation induced impairment of OC differentiation and resorbing activity. Moreover, this is in line with features of the radiation response of other cell types (55, 56).

In contrast, other studies show an enhancement of OC numbers and resorption activities. In this context, a differential impact of low versus higher doses on osteoclastogenesis is discussed, but published results are conflicting (reviewed in (7)), again likely due to the high variability in the study designs as discussed above. However, observation over longer times after irradiation may reconcile these opposed results. In mice exposed to 5 Gy, OC are initially activated, leading to bone loss, but a late onset of long-term depleted OC results in a non-physiological thickening of bone matrix (57), which is discussed as a result of the depletion of marrow-residing progenitors.

Our results suggest that radiation damage induced in an early stage of OC differentiation can lead to an impaired fusion process during terminal differentiation. Scoring of established respective markers of mOC (43, 58), showed significantly decreased cell areas relatively late after exposure (**Figure 4A**) and a smaller average number of nuclei per mOC (**Figure 4C**), again for total doses equal or exceeding 2 Gy. Consistent with the observed late effects of radiation exposure in terms of impaired differentiation of mOC, is the nuclear translocation of NFATc1, which was impaired later, also for fractionated dose delivery, tested for 4x 0.5 Gy (**Figure 5**). This result and the key role in osteoclastogenesis (27) underlines the potential involvement of NFATc1 in the impairment of OC differentiation.

In our study, we have focused on the impact of irradiation of monocytes and the subsequent differentiation into OC. However, the cellular environment, i.e. the intercellular communication of OC progenitors, mOC and other cells, such as T-cells is most likely involved. This is known for osteoclastogenesis and the osteo-immunological cross-talk in inflammatory processes (25, 59, 60). Furthermore, it is in line with bone-forming activities in osteoblasts of *in vitro* and *in vivo* irradiated rodents reported for LD-RT (7, 19, 20, 37), and thus anti-inflammatory processes mediated by LD-RT could lead to a restored balance in bone homeostasis (61–63).

Important for osteoclastogenesis are other leukocytes, such as peripheral monocytes, which are relatively radioresistant (64). As different phenotypes of the monocytes determine the ability of OC to differentiate, the question about a potential radiation induced modulation of osteoclastogenesis by changing the phenotype (65) could be subject for further studies. Interestingly, a decreased activation of monocytes has been shown after LD-RT in the interim analysis of our study (36).

We assume that the reduced bone resorption observed *in vitro* after irradiation is relevant for part of the mechanistic basis of LD-RT. Reported data only provide indirect evidence for this assumption, i.e. the improved healing of bone fractures after low dose exposure (20, 21). To check for it, we collected blood and plasma of Calcaneodynia and Achillodynia patients before, during and after LD-RT, quantified immune cells involved in the regulation, and measured established markers of resorption and formation of bone. According to a rough estimation based on physiological and exposure parameters, less than a few percent of the circulating blood cells are irradiated with 0.5 Gy during a standard local, repeated LD-RT treatment (6 fractions à 0.5 Gy). Therefore, one cannot expect a direct impact on circulating blood cells, evoked by irradiation and relevant for bone metabolism. Hence, only an indirect response in peripheral blood cells is conceivable in this case, transmitted by irradiated cells located in the irradiation field and potentially amplified.

A local radiation treatment of bony structures implies the exposure of bone marrow, where bone precursor and immune cells cohabitate. During regulation of bone resorption and formation, both bone precursor and immune cells undergo complex interactions, with the potential to enhance or to shift immune responses (66). A potential indicator is a modified proportion of T helper and regulatory T cells in the peripheral blood (reduced Th17/Treg ratio), which is reported to correlate inversely with bone resorption markers (66, 67). We could not find such a modified proportion in the respective analysis (**Figures 6A–D**). Moreover, the markers indicating local changes in bone resorption were not consistently modified; a significant decrease of CTX (**Figure 6E**) faces an unchanged activity of the bone resorbing enzyme TRAP 5b (**Figure 6F**). In addition, bone resorption activities, assessed in *ex vivo* differentiated OC from LD-RT patients, did not show significant modifications after treatment (**Figure S3**). In view of the diseases of the patients enrolled in the study -Calcaneodynia and Achillodynia- where also bone formation can occur, we checked a respective marker for bone formation (OCN), which turned out to decrease significantly (**Figure 6G**). Taken together, the results do not provide consistent evidence for changes after treatment over time and we consider that the impact of local irradiation is somehow “diluted” in the circulation, in contrast to whole body exposure such as in Radon Spa therapy, where effects were measurable (9, 10).

Thus, our *in vivo* study performed with Calcaneodynia and Achillodynia patients, did not reveal clear indications for a radiation induced inhibition of bone degradation and resorption after LD-RT treatment. On this basis, we cannot infer a general relevance of the *in vitro* observed reduced differentiation and functionality of OC after irradiation for LD-RT. The pain relief reported by patients enrolled in the same study could at least for Calcaneodynia and tendinopathies be due to other modifications, i.e. the reported small but significant changes in the activation of immune cells (36). As for RA, reduced bone degradation after LD-RT has been shown in a preclinical model (37); the goal of further studies is to investigate the relevance of our *in vitro* observations in arthritic patients treated with LD-RT.

We conclude from our *in vitro* study that fractionated low-dose irradiation of human OC progenitors as administered in LD-RT reduces resorbing activity and impairs the terminal differentiation of OC. NFATc1 might be involved in this process. However, additional mechanisms most likely play a role in the cellular environment in bones and joints. The *in vivo* relevance of these results remains to be clearly shown in future studies.

## Data Availability Statement

The raw data supporting the conclusions of this article will be made available by the authors, without undue reservation.

## Ethics Statement

The studies involving human participants were reviewed and approved by Institutional Review Board of the Friedrich-Alexander Universität Erlangen-Nürnberg. The patients/participants provided their written informed consent to participate in this study.

## Author Contributions

CF designed the experiments and contributed to the writing of the paper and the analysis of the data; FR, DE, and AT contributed to the design of the experiments, performed the experiments, analyzed the data, and contributed to writing the article; DK, IW, JM, SB, TF, ML, and TM contributed to performing experiments and data analysis, TF advised on statistical analyses; A-JD, IB, and BF contributed to designing and performing the patient study. All authors read and agreed to the manuscript.

## Funding

This work was in part funded by the Bundesministerium für Bildung und Forschung (BMBF; GREWIS-alpha, 02NUK050A and 02NUK050E). The IMMO-LDRT01 trial is funded by Department of Radiation Oncology at Universitätsklinikum Erlangen.

## Conflict of Interest

The authors declare that the research was conducted in the absence of any commercial or financial relationships that could be construed as a potential conflict of interest.

The reviewer MS declared a shared affiliation with the authors ML and TM to the handling editor at the time of review.

## Publisher’s Note

All claims expressed in this article are solely those of the authors and do not necessarily represent those of their affiliated organizations, or those of the publisher, the editors and the reviewers. Any product that may be evaluated in this article, or claim that may be made by its manufacturer, is not guaranteed or endorsed by the publisher.
