# Supplementary material for: Modulation of Differentiation and Bone Resorbing Activity of Human (Pre-) Osteoclasts After X-Ray Exposure
Source: Front Immunol. 2022 May 4;13:817281. doi: 10.3389/fimmu.2022.817281 (PMC9116137; doi:10.3389/fimmu.2022.817281)
Supplement: Supplementary file 1 [file DataSheet_1.docx]

**Modulation of Differentiation and Bone Resorbing Activity of Human (Pre-) Osteoclasts After X-Ray Exposure**

**Denise Eckert ^1†^, Felicitas Rapp ^1†#^, Ayele Taddese Tsedeke ^1†^, Daniela Kraft ^1^,**

**Isabell Wente ^1^, Jessica Molendowska ^1^, Sidra Basheer ^1^, M. Langhans ^2^, T. Meckel ^2^,**

**Thomas Friedrich ^1^, Anna-Jasmina Donaubauer ^3,4^, Ina Becker ^3,4^, Benjamin Frey ^3,4^**

**and Claudia Fournier1***^1^

^1^ Department of Biophysics, GSI Helmholtzzentrum für Schwerionenforschung, Darmstadt, Germany

^2^ Translational Radiobiology, Department of Radiation Oncology, Universitätsklinikum Erlangen, Friedrich-Alexander-Universität Erlangen-Nürnberg (FAU), Erlangen, Germany

^3^ Department of Radiation Oncology, Universitätsklinikum Erlangen, Friedrich-Alexander-Universität Erlangen-Nürnberg (FAU), Erlangen, Germany

^4^ Department of Macromolecular and Paper Chemistry & Membrane Dynamics, Technical University Darmstadt, Darmstadt, Germany

*** Correspondence:**Prof. Claudia Fournier
[c.fournier@](mailto:c.fournier@)gsi.de

**^+^** these authors contributed equally

^#^present affiliation: Department of Hematology and Oncology, Medical Faculty Mannheim, Heidelberg University, Mannheim, Germany

Supplemental Figures


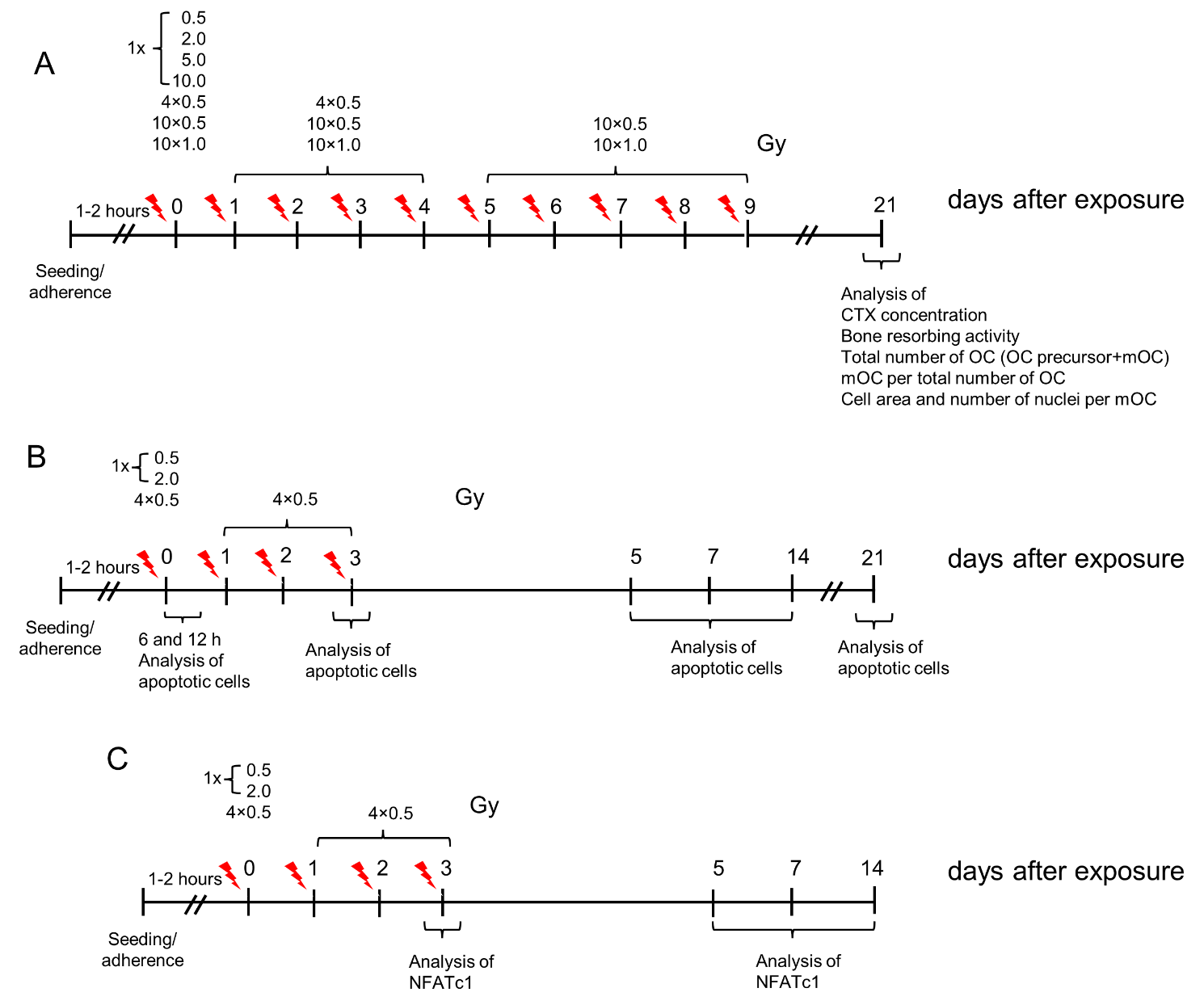


***Fig. S1: Experimental setup.*** *PBMC were attached for 1-2 hours on physiological bone slices. After the adhesion step, attached cells were irradiated with X-irradiation in the presence of growth and differentiation factors (M-CSF, RANKL).* ***(A)*** *For analysis of CTX concentration, bone resorbing activity, the total number of OC (OC precursor + mOC), mOC per total number of OC, cell area and the number of nuclei per mOC after 21 days, attached cells were irradiated with single (0.5, 2, 5 and 10 Gy) and fractionated doses (4 x 0.5, 10 x 0.5 and 10 x 1 Gy in daily intervals).* ***(B****) For analysis of apoptotic frequencies after 6 and 12 hours, and 3, 5, 7, 14 and 21 days, attached cells were irradiated with single (0.5 and 2 Gy) and fractionated doses (4 x 0.5 Gy in daily intervals).* ***(C)*** *For analysis of NFATc1 localization after 3, 5, 7, and 14 days, attached cells were irradiated with single (0.5 and 2 Gy) and fractionated doses (4 x 0.5 Gy in daily intervals).*


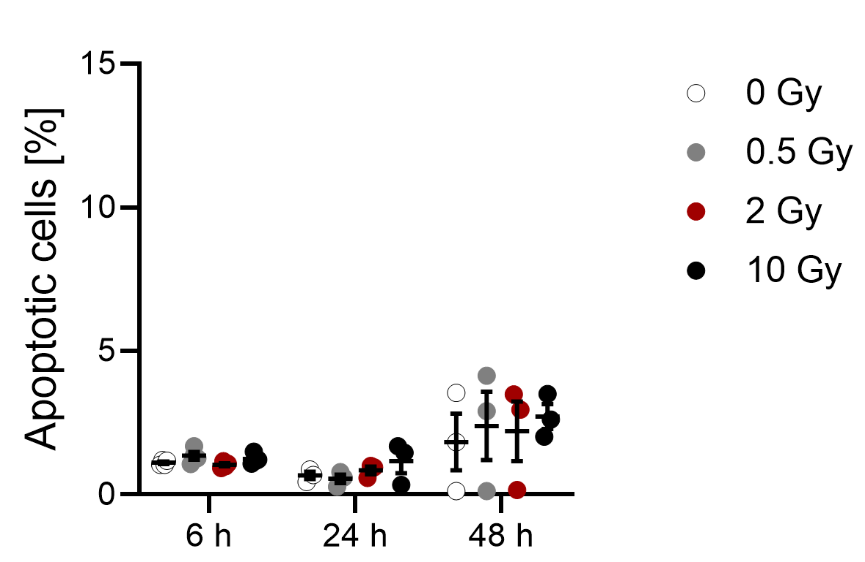


***Fig. S2: Apoptosis in monocytes occursat a very low level after X-irradiation:*** *Monocytes were irradiated with single doses (0.5, 2, and 10 Gy) of X-rays, and incubated without growth and differentiation factors (M-CSF, RANKL) for 6, 24, and 48 hours. Afterward, the cells were washed (500 ×g, 8 min) with PBS and detached with Accutase. Then, monocytes were stained with Annexin-V (FITC, clone, #556419, BD Biosciences, Heidelberg, Germany) for 10 min at 4 °C. Subsequently, monocytes were washed twice with PBS (500 ×g, 8 min). Finally, the fraction of Annexin-V^+^ monocytes was measured with flow cytometry (FACSDiva, BD Biosciences, Heidelberg, Germany). Monocytes were determined in the SSC/FSC dot plot. After exclusion of doublets, Annexin-V-FITC positive cells were classified as apoptotic cells.*

*Significance was tested with one-way ANOVA for normal distributed data. Error bars are reported as the mean ± SEM; N=1-2.*


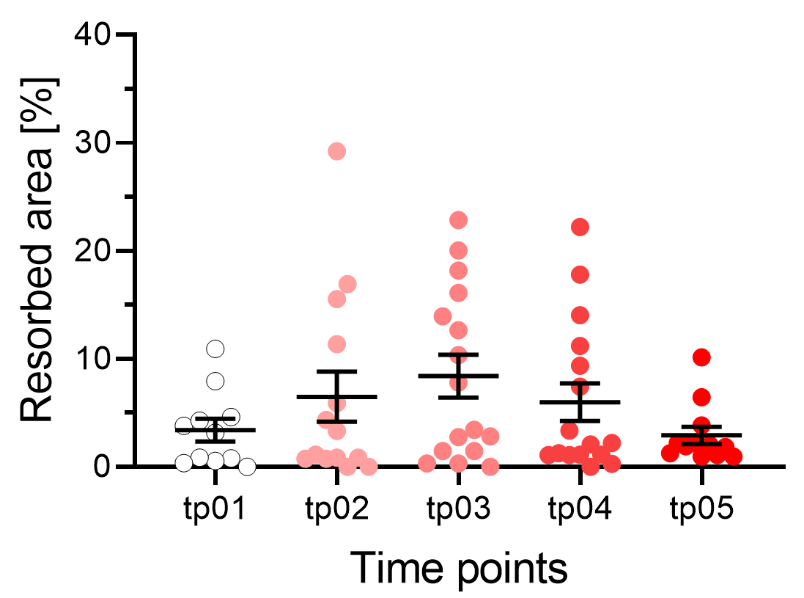


***Fig. S3: Bone resorbing activity is not significantly changed after LDRT in patients.*** *Monocytes isolated from patients were attached on bone slices and cultivated ex vivo in the presence of growth and differentiation factors (M-CSF, RANKL) for 21 days. Resorbed area on bone slices was measured for several time points (tp) before, during and after therapy by Toluidine Blue staining as described. Bone resorption is expressed as resorbed bone area in percent of total bone.*

*Significance was tested with Kruskal-Wallis for non-normal distributed data. Error bars are reported as the mean ± SEM; N=11-16.*
